# Alignment of angular velocity sensors for a vestibular prosthesis

**DOI:** 10.1186/1743-0003-9-14

**Published:** 2012-02-13

**Authors:** Jack DiGiovanna, Jacopo Carpaneto, Silvestro Micera, Daniel M Merfeld

**Affiliations:** 1 Neuroprosthetics Control Group - Automatic Control Lab, ETH Zurich, Physikstrasse 3, ETL k24, 8092 Zürich, Switzerland; 2 BioRobotics Institute, Scuola Superiore Sant'Anna, Pisa, Italy; 3 Jenks Vestibular Physiology Lab, Massachusetts Ear & Eye Infirmary, Boston, USA; 4 Otology and Laryngology, Harvard Medical School, Boston, USA

**Keywords:** Vestibular prosthetics, sensor alignment, neuro-prosthetics

## Abstract

Vestibular prosthetics transmit angular velocities to the nervous system via electrical stimulation. Head-fixed gyroscopes measure angular motion, but the gyroscope coordinate system will not be coincident with the sensory organs the prosthetic replaces. Here we show a simple calibration method to align gyroscope measurements with the anatomical coordinate system. We benchmarked the method with simulated movements and obtain proof-of-concept with one healthy subject. The method was robust to misalignment, required little data, and minimal processing.

## Background

Imagine waking up one morning, opening your eyes, and seeing your bedroom rotated 90 degrees. Suddenly, instead of the ceiling appearing above you, it is to your right. Would you be able to function? Could you adapt to such a sensory misalignment? In the long-term, the answer is yes. The brain is capable of resolving misalignment between normal and received sensory information through the mechanism of neural plasticity. Gonshor and Mevill Jones showed one dramatic example of such plasticity in their work using prism glasses [1]. These glasses inverted subjects' view of the world (e.g. right is left), but over days subjects adjusted their vestibulo-ocular (VOR) reflexes; by 18 days the VOR had reversed to match visual information. However, such plasticity is neither immediate nor free. Subjects in preliminary trials reported "rapid and severe nausea" [1] and VOR following adaptation to vision reversal never fully mimicked normal responses.

Vestibular prosthetics transmit sensory information to the brain, but they also can induce sensory misalignment. These prosthetics should (partially) replace vestibular organs, which sense gravity, linear acceleration, and angular acceleration (rotation) of the head. Normally, information from these organs is transmitted to the brain where it is fused with visual and other sensory inputs to yield spatial orientation and on-going movements of the body [2,3]. Vestibular prosthetics aim to address some symptoms of vestibular dysfunction including: spatial disorientation, postural instability, self-motion perception deficits, visual blurring during head motion ("oscillopsia") due to loss of VOR, and chronic disequilibrium. However, there can be misalignment between the information transmitted by the prosthetic and information formerly provided by the damaged sensory organs. Two existing approaches solve this problem: 1) align the prosthetic sensors to the user's anatomy during the implantation surgery; 2) allow brain plasticity to correct any misalignment of the implanted prosthetic. These approaches are not exclusive; in fact 2) will happen regardless of how or if 1) was completed. The required plasticity may not be as extreme as for prisms [1], but time and energy are still required.

We suggest that providing an initial condition that is close to natural semicircular canal SCC information will decrease learning time (effort) for the brain and also may increase mapping accuracy. This can be accomplished by approach 1) above, but it requires additional efforts by the surgical team and lengthens surgery time for the patient. We propose an alternative approach that will achieve the same ends as surgical prosthetic alignment but can be completed outside of the operating room. Our approach uses a simple sensor that is temporarily secured to the head via a bite-bar (a device commonly used to hold film during a dental x-ray). Measurements from this bite-bar are collected synchronously with the vestibular prosthetic sensor. The calibration method we propose finds the matrix necessary to align prosthetic sensor measurements to the bite-bar (also to the head by proxy); then using average anatomical positions (or medical imaging techniques) the sensor can be further aligned to the vestibular organs. Our calibration method is not restricted by time under anaesthesia and can take advantage of advanced imaging techniques. We will show that it is possible to replace the surgical alignment with our method; additionally, this will produce more accurate alignments of the vestibular prosthetic sensor to the damaged vestibular organs.

**Figure 1:**
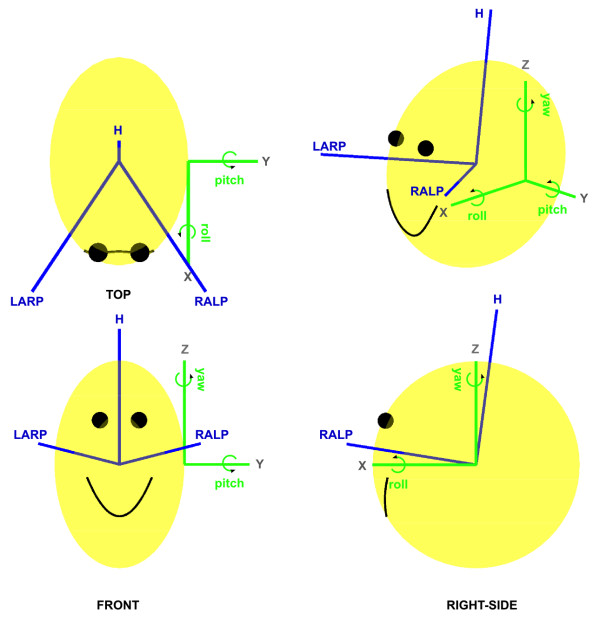
**Multiple sensory coordinate frames in a vestibular prosthetic**. The approximate semi-circular canal (CANAL) coordinate frame has blue axes and labels based on canal names (LARP: left anterior - right posterior, RALP: right anterior - left posterior, H: horizontal). The prosthetic sensor (HMMS) axes are green with grey axes labels. HMMS rotations are also labeled (green), e.g. pitch is a rotation about the <Y> axis. The HEAD coordinate system is based on REID coordinates and uses the same axes labels (grey) as the HMMS. However, HEAD axes are *not *shown here to avoid clutter. The HEAD frame is *aligned *with the HMMS but *coincident *with CANAL.

The sensory misalignment problem in vestibular prosthetics is summarized here. Vestibular research has provided a framework for addressing vestibular disorders related to head rotations [4-10]. Specifically, electrically stimulating vestibular neurons (VIII nerve afferents activated via an electrode proximal to the ampullary cupula) can mimic the natural (healthy) encoding of angular velocity [5,8,10,11]. Angular velocity can be measured by electrical gyroscopes (gyros); attaching three gyros to the skull (with one gyro aligned to each semi-circular canal) would mimic physiological detection of head rotation^i^. It is possible to rigidly affix orthogonal gyros to the skull, e.g. on the mastoid process (a common attachment point for cochlear implants [12]). However, surgical and other complexities can create sensory misalignment discussed in prior paragraphs (also see Figure 1). Furthermore, it is not straightforward to accurately measure the orientation of the gyros relative to the canal geometry during (or after) implantation.

Aligning different sensor systems was an early problem for naval weapons systems that was addressed with strap-down gyro triads [13]. Gyro digital outputs were processed by a PC using a least-squares optimization to find nine entries of the rotation matrix, which accounted for sensor misalignment. Vestibular prosthetics have a static displacement between the sensors (gyros and SCC); here we are only concerned with angular velocities. Thus finding a rotation matrix between the sensors will align them. This problem was anticipated and a practical solution patented in 2008 [14].

In this paper we will develop and test a simple optimization algorithm to find a rotation matrix for vestibular prosthetic sensor alignment. The algorithm transforms artificial sensor measurements (green coordinate system in Figure 1) into a physiologically appropriate coordinate system (e.g. blue coordinate system in Figure 1). Robust performance is demonstrated in simulations tailored to anticipated implementation issues, where simulated sensor readings were generated based on actual head measurements. Additionally, this method creates a more accurate signal than surgical alignment. We performed a proof-of-concept alignment of real sensor recordings (human data) to confirm simulations of independent sensor noise effects and test bite-bar sensor approximation of skull movements. Finally, based on the method's strengths and weakness, we proposed a clinical protocol and implementation to rapidly and accurately calibrate a vestibular prosthetic sensor for human patients.

## Methods

### Optimization

A rotation matrix describes the transformation between two coordinate frames that are rotated with respect to one another. Given a rotation matrix *R*, it is possible to calculate a measurement in rotated frame *a *from the measurement in coordinate frame *b*, if the measurements span all dimensions:

(1) $$\vec{a}=R\vec{b}$$

Specifically, for a three dimensional measurement (where ***a ***= [*a*_*x *_*a*_*y *_*a*_*z*_]^T^), there are nine entries in *R *(3 × 3). It is possible to calculate *R *for a specific sequence of rotations between frames *a *and *b*, one common method for this is Euler angles [15]. Equation 2 shows calculation of *R *from Euler rotation angles α, β, and γ for a ZXY rotation order^ii^, where *c *and *s *are cosine and sine, respectively.

(2) $$\begin{array}{c} R_{Z^{\prime }X^{\prime }Y^{\prime }}(\alpha ,\beta ,\gamma )=\\ \left[\begin{array}{ccc} -s(\alpha )s(\beta )s(\gamma )+c(\alpha )c(\gamma ) & -s(\alpha )c(\beta ) & s(\alpha )s(\beta )c(\gamma )+c(\alpha )s(\gamma )\\ c(\alpha )s(\beta )s(\gamma )+s(\alpha )c(\gamma ) & c(\alpha )c(\beta ) & -c(\alpha )s(\beta )c(\gamma )+s(\alpha )s(\gamma )\\ -c(\beta )s(\gamma ) & s(\beta ) & c(\beta )c(\gamma ) \end{array}\right] \end{array}$$

In the vestibular prosthetic, **both **the rotation angles and the velocity measurements in the canal coordinate system (*a *in Eqn. 1) **are unknown**. We first demonstrated we could solve for R in an *ideal *calibration environment where measurements in both coordinate systems were available but rotation angles between the two coordinates were unknown. This calibration is similar to prior literature that used a linear least squares optimization to find the nine elements of *R *[11,13] by minimizing *J:*

(3) $$J\left(R\right)=\text{mean}\left(\text{mean}\left({\left(\vec{a}-R\vec{b}\right)}^{2}\right)\right)$$

We used a similar optimization but restricted *R *to Euler rotations having just three degrees of freedom and computed RMS error (instead of MSE because typical errors were less than one):

(4) $$\begin{array}{c} \;\;\;\;J\left(\alpha ,\beta ,\gamma \right)=\\ \text{mean}\left(\text{mean}\left(\text{abs}{\left(\vec{a}-R\left(\alpha ,\beta ,\gamma \right)\vec{b}\right)}^{2}\right)\right) \end{array}$$

A sequential quadratic programming algorithm (Matlab's fmincon function) was used to optimize the rotation angles (α, *β, γ*) given angular velocity measurements in the two coordinate systems while accounting for the presence of constraints (i.e. physiological limits and trigonometric functions in the rotation matrix). To decrease the probability of selecting a local minimum, we repeated the optimization from ten random, but widely dispersed (+/- 90°), initial conditions (angles).

Our approach has fewer parameters (3 vs. 9) - thus reducing the number of training samples - and it ensures orthogonality. However, the approach in Eqn. 3 has lower computational complexity (e.g., no trigonometric functions). Since our calculations can be performed off-line and do not require long time series, computational complexity was not a paramount concern.

### Coordinate Systems

The four coordinate systems to be used in this analysis are defined here. Figure 1 shows the Head-Mounted Motion Sensor (HMMS) coordinate system in green. The HMMS is aligned to the HEAD coordinate system (but the HMMS origin is translated from mid-skull). The origin of the HEAD coordinates is coincident with the origin of the REID coordinates [16]. The semi-circular canal (CANAL) coordinates are shown in blue. The origin of the CANAL coordinates is coincident with origin of HEAD, but rotated (fixed-angle rotation) -19.9° about <Y> and +43.45° about <Z> to *roughly *approximate the average human CANAL orientation found in [16]. (Of course, if desired, imaging or other methods could be used to find the canal orientation for each patient. This orientation would define patient-specific CANAL coordinates.) The final coordinate system is the Bite-Bar Sensor (BBS) coordinates that will be aligned with HEAD but the origin will not be coincident (BBS is not shown in Figure 1). Our main goal is to rotate HMMS measurements to align them with CANAL coordinates. The BBS and HEAD coordinates are crucial intermediaries to attain this goal.

### Simulation testing

We used simulations to answer questions about the alignment procedure that would not be practical/ethical to address with human experiments:

1) Does HMMS alignment via the BBS provide any advantage over manual alignment of the HMMS and HEAD during surgery?

2) How does signal error change as a function of HMMS misalignment?

3) How does independent, additive sensor noise affect the alignment algorithm?

Additionally, since the optimization (Eqn. 4) is across alignment angles rather than velocities it is not necessary for training set velocities to cover the full range of velocities to be experienced. However, we wanted to clearly demonstrate a potentially clinically relevant feature with the comparison:

4) Can slow movements be used for training the alignment algorithm such that it can generalize to faster movements?

We used 45 s of recorded human movements (details in next sections) as "true" HEAD measurements. Rotating these HEAD measurements (as detailed in the prior section) created average CANAL measurements. We assumed that manual alignment of the HMMS and HEAD during surgery (*surgical alignment*) would be accurate (zero-mean) but could not be perfectly precise (2° standard deviation) yielding normally distributed (zero-mean; 2° standard deviation) alignment errors. The assumption of accurate alignment is not essential for our method, since the algorithm will correct for any HMMS: HEAD misalignment. The assumption is made to provide a baseline to challenge the algorithm to yield performance equal to or better than that achieved surgically. In summary, such an accurate surgical alignment would generate a $\hat{R}$ (that in the absence of any errors is the identity matrix) with small surgical alignment errors *e*_*i *_added to actual rotation angles (*α, β, γ*) (here equal zero - compare with Eqn. 2).

(5) $$\hat{R}=R_{Z^{\prime }X^{\prime }Y^{\prime }}\left(e_{1},e_{2},e_{3}\right)$$

Given this rotation matrix, it was possible rotate our HEAD measurements into the HMMS coordinates:

(6) $$\omega_{\text{HMMS}}={\hat{R}}_{\text{HEAD}}^{\text{HMMS}}\cdot \omega_{\text{HEAD}}$$

In Equation 6 we add a subscript and superscript to $\hat{R}$ to specify the original and rotated coordinate frame respectively. HMMS measurements will be more or less aligned to the HEAD depending on surgical alignment errors (Eqn. 5). HMMS can then be rotated into CANAL coordinates:

(7) $${\hat{\omega }}_{\text{CANAL}}=R_{\text{HEAD}}^{\text{CANAL}}\cdot \omega_{\text{HMMS}}$$

Equation 7 is an estimation of ω_CANAL_, we quantified the error ($\omega -\hat{\omega }$) of this approximation with two metrics: RMS error (Eqn. 8) and point-to-point error (Eqn. 9). In equations 8 and 9, ω is the actual velocity, $\hat{\omega }$ is the predicted velocity, and *T *is signal length. Equation 9 also disregards samples where the actual velocity magnitude was less than 2.09°, which is three times the RMS sensor measurement at rest (assumed noise).

(8) $$\text{erro}{\text{r}}_{\text{RMS}}=\frac{1}{3}\overset{3}{\underset{i=1}{\sum }}\frac{1}{T}\overset{T}{\underset{t=1}{\sum }}\left|\omega_{i,t}-{\hat{\omega }}_{i,t}\right|$$

(9) $$\begin{array}{c} \text{erro}{\text{r}}_{\text{p - t - p}}=\frac{1}{3}\sum_{i=1}^{3}\frac{1}{T}\sum_{t=1}^{T}\left|\frac{\omega_{{\text{d}}_{i,t}}-\omega_{{\text{y}}_{i,t}}}{\omega_{{\text{d}}_{i,t}}}\right|\\ \;\;\;\;\;\forall \omega_{{\text{d}}_{i,t}}>3\omega_{\text{rest}} \end{array}$$

We assumed a BBS would be better aligned with HEAD because it can be done before the vestibular implant surgery via imaging technologies (e.g. x-ray, CT) and is not limited by surgery time or mastoid geometry. Specifically, the BBS would have normally distributed (zero-mean; 0.5° standard deviation) alignments errors (*e*_*i *_in the approximation (Eqn. 5) of *R *between HEAD and BBS). Though the BBS and surgical alignment errors are approximate, we have chosen values that are about right. Certainly, all the same technologies that might be available for a surgical alignment of the sensors are available for this post-surgical alignment - without the constraints present during surgery. Hence, for these simulations, what is important and justifiable is that the precision of the BBS alignment be assumed better than the precision of surgical HMMS alignment.

In exchange for the time invested in BBS alignment, there would be only minimal effort in aligning the HMMS during surgery. Specifically, we allowed HMMS alignment errors to increase by a factor of 50 to be zero-mean; 100° standard deviation (*e*_*i *_in the approximation (Eqn. 5) of *R *between HEAD and HMMS). Despite this large HMMS-HEAD misalignment, HMMS signals can still be rotated to CANAL coordinates via the BBS.

(10) $${\hat{\omega }}_{\text{CANAL}}=R_{\text{HEAD}}^{\text{CANAL}}\cdot R_{\text{HMMS}}^{\text{BBS}}\cdot \omega_{\text{HMMS}}$$

In Eqn. 10, the **R **is the optimized rotation matrix calculated using Eqn. 4 (where *a *= BBS and *b *= HMMS). Errors in this alignment method are quantified in the same fashion as surgical (see eqns. 8 and 9)

To compare both error metrics over a distribution of possible surgical or BBS misalignments, we took 10,000 samples from each distribution and calculated the corresponding CANAL signals via Eqn. 7 and Eqn. 10. This will answer question 1). Question 2) was investigated more directly by setting the error terms in Eqn. 5 (errors for each axis (*e*_*i*_) were set equal and varied from -45° to 45°) and calculating the corresponding CANAL signals via Eqn. 7. Question 3) is addressed by independently injecting additive measurement noise in the BBS and HMMS measurements. Equation 11 describes "noisy" signals where added noise (ν ~N(0,1)) was multiplied by a percentage (*p*) of RMS angular velocity for each axis (*i*). The alignment error (Eqn. 12) was checked over various error percentages (*p*) and quantified with eqns. 8 and 9. To demonstrate that an alignment trained with slow movements will generalize to faster movements we used the signals in Eqn. 12 without excess noise (*p *= 0) and compared using slow or representative speed training sets.

(11) $${\tilde{\omega }}_{i}=\omega_{i}+\nu \cdot p\cdot \text{RMS(}\omega_{i}\text{)}$$

(12) $$\text{error}={\tilde{\omega }}_{\text{BBS}}-R_{\text{HMMS}}^{\text{BBS}}\cdot {\tilde{\omega }}_{\text{HMMS}}$$

### Sensors

Multiple sensors were used for the proof-of-concept human experiments to compare angular velocity measurements in HEAD, BBS, and HMMS coordinate systems. An optic motion capture system (Vicon 460, Vicon Motion Systems, Oxford UK) was used precisely measure the position of the skull over time. Five cameras (Vicon M2 (Vicon Motion Systems, Oxford UK)) were placed around the subject with reflective markers attached to the skin over the skull providing spatially accurate (< 0.8 mm error) marker positions at 100 Hz resolution. Given these measurements, it was possible to calculate angular velocities in HEAD coordinates (details in next section). As shown in the prior section, HEAD measurements can be rotated into CANAL coordinates via the matrices in [16] or patient-specific coordinates using a CT scan.

While motion capture systems are often used in research laboratories, it is less common to find them in clinical settings. The systems are expensive; require a relatively large operating space; and trained personnel. Thus, we proposed a BBS as a replacement for both surgical alignment of the HMMS to HEAD and direct HEAD measurements. The BBS is a much smaller, easier to operate, and less expensive. Specifically, we mounted two 2D gyroscopes (LPR5150AL and LPY5150AL, STMicroelectronics) orthogonally to a common dental "bite-bar" (designed to hold film at a specific distance from the x-ray camera) to measure angular velocities of the head. These low-power, dual-axis, micromachined gyros are capable of measuring angular rate along pitch and roll (LPR5150AL) or along pitch and yaw (LPY5150AL) axes with a full scale of ±1500°/s and with a -3 dB bandwidth up to 140 Hz. Our assumption was that the molars are approximately perpendicular to the 'pitch' <Y> axis (interaural) of the head and the geometry of the bite bar created surfaces aligned to both the pitch and 'yaw' <Z> axes. By design, the orthogonal gyros mounted on these surfaces would be approximately parallel to the pitch, roll, and yaw axes of the head. This complete device is the BBS.

Finally, we needed a device to mimic the Head-Mounted Motion Sensors (HMMS) that would be present in vestibular prostheses. To address this, a commercially available inertial system (MTx by xSens) was rigidly attached to the subject's head. The MTx uses gyros, accelerometers, and magnetometers to determine angular rotations, orientation, and acceleration [17].

### Calculating angular velocity

In the prior section we introduced three sensors (Vicon markers, bite-bar system, and MTx) that provide angular velocity in the HEAD, BBS, and HMMS coordinate systems respectively. The MTx system calculates angular velocity internally; thus HMMS measurements were read-out directly. Similarly, the BBS directly measures angular velocity and outputs a voltage for each axis. This voltage was converted to degrees per second using the manufacturer's data-sheet and a calibration voltage to find BBS measurements. On the other hand, Vicon measures marker positions; thus a transformation was required to get HEAD angular velocities. Markers were used to determine axes for the HEAD segment (specifically forehead and left and right ear markers). Given these axes, angular velocity was calculated:

1. Calculate HEAD *origin *as mean value of three markers [*left_ear, right_ear, forehead*]

2. Define **<y>**as (*left_ear *- *right_ear*)/norm (*left_ear *- *right_ear*)

3. Define **<aux_x>**as (*forehead *- *origin*)/norm (*forehead *- *origin*)

4. Define **<z>**as **<aux_x>**× **<y>**(where × is the cross product)

5. Define **<x>**as **<y>**× **<z>**

6. Create **R **= [**<x> <y> <z>**]

This pseudo-code is applied for each sample; it creates an *R *matrix for every time step. Differentiating *R *yields the angular velocity tensor *W *(Eqn. 13); the components of this tensor (Eqn. 14) are the angular velocity of the head at each time step.

(13) $$W=\frac{dR}{dt}R$$

(14) $$W\left(t\right)=\left(\begin{array}{ccc} 0 & -\omega_{z}\left(t\right) & \omega_{y}\left(t\right)\\ \omega_{z}\left(t\right) & 0 & -\omega_{x}\left(t\right)\\ -\omega_{y}\left(t\right) & \omega_{x}\left(t\right) & 0 \end{array}\right)$$

This technique was validated with artificial data (sinusoidal angular velocity at 50°/s). We found perfect correlation (R^2 ^= 1) between the angular velocities applied to the data and those found using Eqns. 13 and 14. Additionally, we applied white noise equivalent to the Vicon camera calibration accuracy and then a low-pass filter (5^th ^order Butterworth, f_c _= 10 Hz) similar to the post-processing in Vicon software (Workstation). This measurement noise reduces R^2 ^to 0.9706. This should be considered the upper bound of performance for HEAD measurements.

### Recording protocol

**Figure 2:**
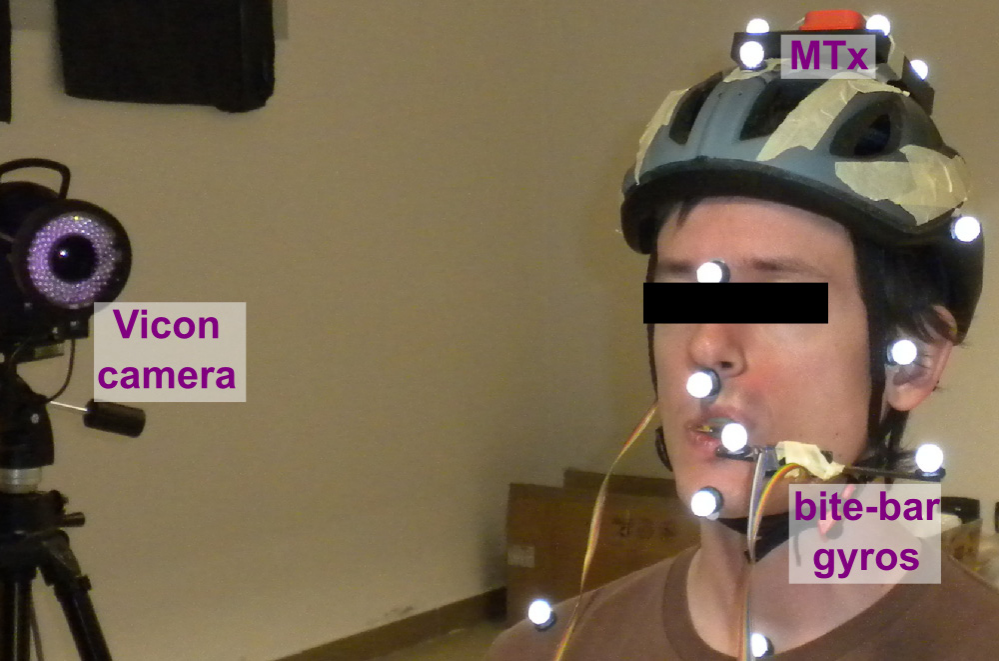
**Instrumented test subject**. Motion capture markers (white balls) are placed near facial landmarks. The helmet was tightened to avoid shifting relative to the skull. MTx measurement system (orange box) is mounted to helmet as a proxy for artificial vestibular sensors. Wires connecting to MTx are on subject's right. Subject was instructed to bite hard on bar to keep it stable. Bite-bar gyro and wiring on subject's left.

The protocol was designed to answer the question, *"Could we calibrate the alignment of vestibular prosthetic sensors (HMMS) with the SCC using simple head rotations*?" If so, a secondary, practical question was "*Are bite-bar gyros (BBS) sufficient to measure head (HEAD) rotations or are they too noisy*?" **To investigate both questions, we tested all three sensors concurrently**. Specifically, the subject was instrumented with the MTx system, motion capture markers, and bite-bar gyros (see Figure 2). To simulate worst-case motion artifacts, the subject bit down on the bite-bar without custom-fitted dental material. (This means BBS axes were only roughly aligned to HEAD.)

We used the following protocol to simulate calibration of the HMMS component of a vestibular prosthetic. (Note, here we evaluate calibration via BBS or HEAD measurements, i.e. only step 3 or 4 would be necessary in a real application.)

1) Have subject sit in a chair in the middle of recording area.

2) Mount vestibular prosthetic sensors (HMMS) to subject.

• Normally this would already be accomplished in surgery. Here we used a tight-fitting bike helmet with a rigidly attached MTx for healthy subjects.

3) Attach reflective markers to subject (for HEAD measurements)

• Increasing the number of markers slightly improves calculation of rotation angles. However, exact placement at specific landmarks is not necessary.

4) Align bite-bar with interaural axis and have subject bite down firmly to keep it stable (for BBS measurements)

5) Begin recording Vicon, BBS, and MTx sensor measurements synchronously

6) Ask subject to move head for ~30 s completing the following movements:

• Range of motion for each rotation axis slowly then quickly. (Referred to as *Fast *and *Slow ROM*.)

• Normal (subject unrestricted) movements slowly then quickly. (Referred to as *Fast *and *Slow Explore*.)

• Briefly (~2 s) rest between each movement type

To ensure we would have sufficient data for various analyses, we collected more data than necessary (in steps 3 and 6). To validate the robustness found in artificial data, we tried multiple sensor alignments (step 2). One healthy male (study author, age 29, height 183 cm) subject performed this recording protocol.

## Results and discussion

### Simulations

**Figure 3:**
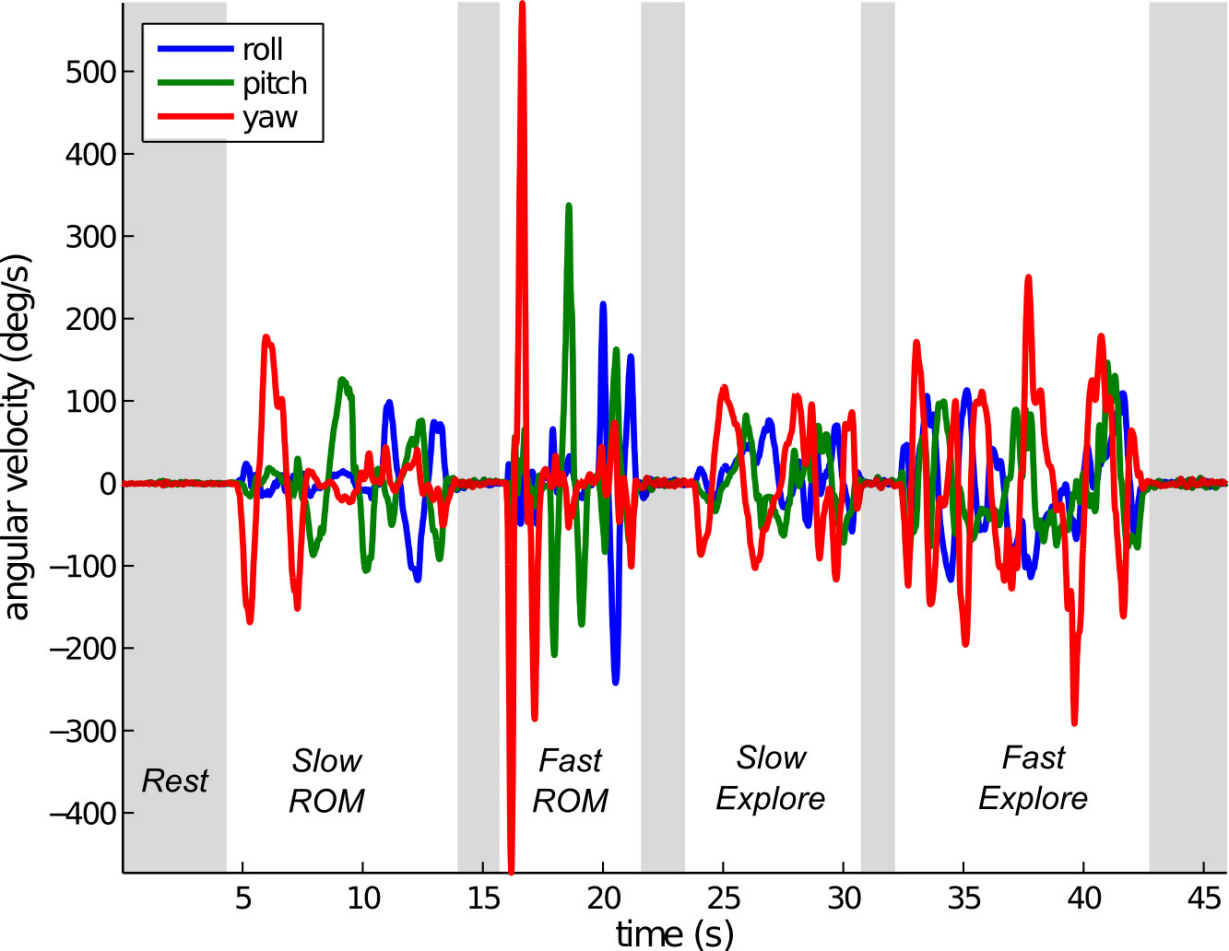
**HEAD angular velocity**. Angular velocity recording during a human trial consisting of Slow ROM, Fast ROM, Slow Explore, and Fast Explore phases. This data was collected using the MTx approximately aligned to HEAD. Here we *define *these data as HEAD and simulate measurements in the other coordinate systems.

**Figure 4:**
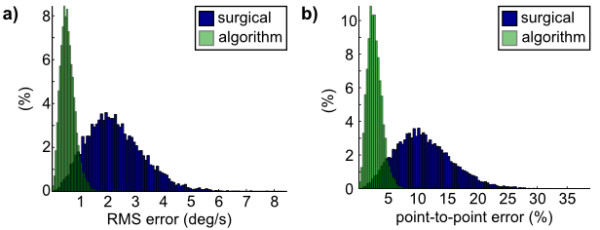
**Surgical vs. algorithm alignment**. Simulated alignments of the HMMS to CANAL based on random initial misalignments (N = 10,000). Two techniques were compared: surgical alignment (blue) of the HMMS-HEAD or algorithm alignment (green) of the HMMS-HEAD via the BBS. (a) Probability density function of RMS error shows lower mean error for algorithm (0.58°/s) compared to surgical (2.29°/s) alignment. (b) Probability density function of point-to-point error also shows lower mean error for algorithm (2.21%) compared to surgical (11.04%) alignment.

Using simulations, we extensively tested optimization algorithm performance using the HEAD measurements shown in Figure 3 as described in *Methods*. First, we compared the proposed alignment of *HMMS to CANAL via a BBS *by simulating direct surgical alignment of *HMMS to HEAD *using 10,000 HMMS and BBS alignments (each representing a hypothetical HMMS attachment to a patient's skull) for each method (randomly sampling the alignment distributions in Eqn. 5). Figure 4 shows both error metrics have a lower mean and smaller variance for the proposed alignment relative to surgical alignment. This holds despite the fact the HMMS-HEAD misalignment had 50× higher variance (relative to surgical alignment) when a BBS was used.

**Figure 5:**
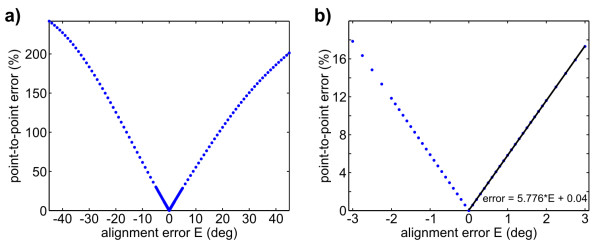
**Point-to-point error over surgical misalignment**. Comparison of HMMS-CANAL errors introduced by a range of surgical alignment errors. (a) Alignment error was added to all rotation angles in Eqn. 5. (b) Zooming in on the same errors over a range of 3° and fitting a curve here shows that point-to-point velocity error increases nearly 6× faster than angular misalignment.

A follow-up question was how much more accurate surgical alignment would need to be in order to reduce error to similar levels as the BBS alignment. We set the errors for each axis (*e*_*i*_) equal and varied them from -45° to 45°. Figure 5 shows the rapid increase in point-to-point error with misalignment. The inset zooms in on error from -3° to 3°, a linear fit in that region shows that the point-to-point error (%) increases almost 6× faster than alignment error (°). Thus error in a vestibular prosthetic would be very sensitive to any surgical misalignment if a BBS was not used. However, notice in Figure 4 that the error remained low (mean 2.2%) when a BBS was used even with HMMS-HEAD misalignment being ~N(0, 100).

**Figure 6:**
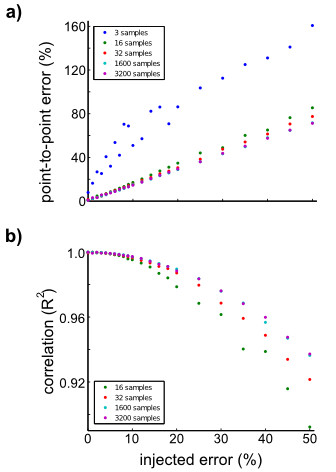
**Sensor misalignment with injected independent noise**. Additive white noise progressively degraded sensor alignment for a variety of training set sizes. (a) Point-to-point error increases with the percentage of error. Increasing the training set size from 3 to 16 samples (300-1600 ms) greatly reduced to slope of increase. However, further increases had minor effect, e.g. results for 1600 and 3200 samples overlap. (b) Correlation revealed a similar trend (3 sample results removed here to highlight the differences in the other training set sizes). Regardless of training set size, the testing sets were the same separate segment (1200 samples).

Clearly the proposed BBS method is superior to any surgical alignment with more than 0.4° standard deviation alignment error (*e*_*i *_). However, this method does require an additional sensor (i.e. BBS); this means there will be two independent measurement noises (in the BBS and HMMS). In the prior example there was measurement noise in the HEAD measurements (from an MTx) that was rotated into the simulated HMMS and BBS sensors. Thus realistic noise was present, but only from a single source. In Figure 6 we evaluated sensor misalignment (Eqn. 12) across percentages of noise (*p *in Eqn. 11). Independently injecting 10% noise on both sensors resulted in approximately 14% point-to-point error and reduced the correlation (R^2^) to approximately 0.997 (for any training set ≥ 1600 samples). This error was an extreme example of measurement noise, especially for large *p*, because the error-injected signals were not low-pass filtered before alignment. This filtering would have reduced the noise effects; such filtering is a standard procedure for all sensors used in our experiments.

A common approach to minimize the effect of zero-mean noise sources is to use longer data segments because the optimizer finds the mean solution. In Figure 6 errors initially decrease with additional training samples for both metrics; however, this effect quickly saturates (i.e. above 1600 samples (16 s)). This saturation is reasonable because of the structure of the error. In Eqn. 12, the **R **component should improve with additional samples but the desired signal (${\hat{\omega }}_{\text{BBS}}$) remains corrupted by noise. Thus the overall room for improvement is limited.

**Figure 7:**
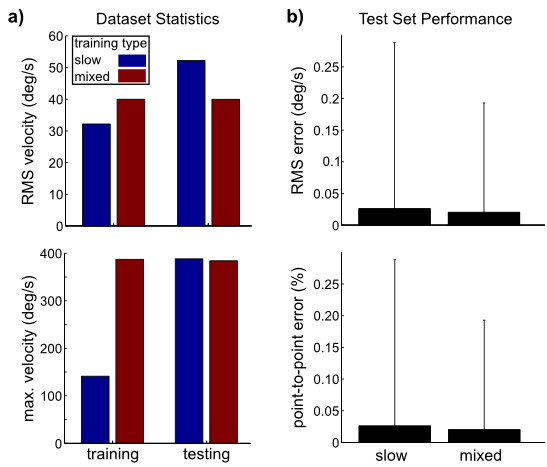
**Algorithm alignment for slow or representative training sets**. To demonstrate that algorithm is insensitive to dataset statistics we used two different training sets: slow (blue) and mixed (red). (a) Training set statistics show the slow training set does not contain the velocities observed in the testing set. (b) Both error metrics did not show a significant difference between the two training sets.

Finally, we demonstrated the feature that slower angular velocities can be used for training to find an **R **that will generalize to faster angular velocities. Specifically, we used the *Slow ROM *segment for training and check generalization during *Fast ROM *(see Figure 3 for segment labels). Figure 7 shows this error was not significantly different than if a training set was randomly selected from both segments. Using slower and smaller rotations is potentially a desirable feature for clinical application.

### Alignment of sensor pairs

Through the simulations above, we have shown the advantages of aligning the HMMS to the HEAD via a BBS rather than manual HMMS-HEAD alignment during surgery. *These advantages form the primary purpose of this study*. However, to demonstrate that the solution is practical, we also conducted a single proof-of-concept experiment with a human subject. In the experiment, we investigate if the BBS provided stable HEAD angular velocities measurements during actual movements. If there is no additional noise (e.g. bite-bar slip) during the experiment, the simulations have shown that it is possible to align the HMMS and BBS sensors with low error. For comparison, we used the Vicon system to directly measure head position and calculate HEAD angular velocities via Eqn. 13. Then we benchmarked HMMS-BBS against HMMS-HEAD alignment.

**Figure 8:**
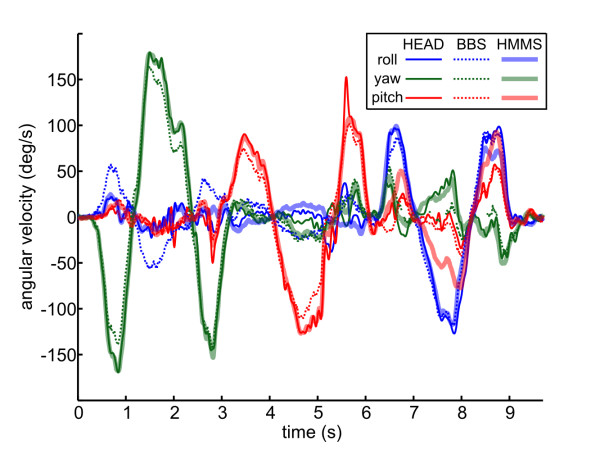
**Comparison of angular velocities measurements during *slow ROM***. HEAD (solid), BBS (dotted), and HMMS (dashed) measurements are shown (HMMS measurement obscured by overlap with HEAD). In this trial all sensors were physically aligned. Mean R^2^: HEAD-HMMS = 0.93; BBS-HMMS = 0.89; HEAD-BBS = 0.89.

In this section, angular velocities were measured in the HMMS, HEAD, and BBS by the MTx, Vicon, and bite-bar gyros respectively. An example is shown in Figure 8 for a Slow ROM segment for all three coordinate systems where all sensors were manually brought into approximate alignment (trial 1).

Before each successive *trial *(steps 4-6 of the recording protocol, see *Methods*) we introduced a progressively larger misalignment by rotating the MTx mounting plate (point of rigid attachment to the helmet) clockwise (average increments of 67 deg); this created a majority of misalignment in the HMMS *yaw *axis. For all trials, we used the optimization described in *Methods-Optimization *to solve for *R *using the cost defined in Eqn. 4. Separate optimizations were performed, one to align HMMS to HEAD; another to align HMMS to BBS. The HMMS was a common sensor, thus any HMMS measurement noise was common to both optimizations.

**Figure 9:**
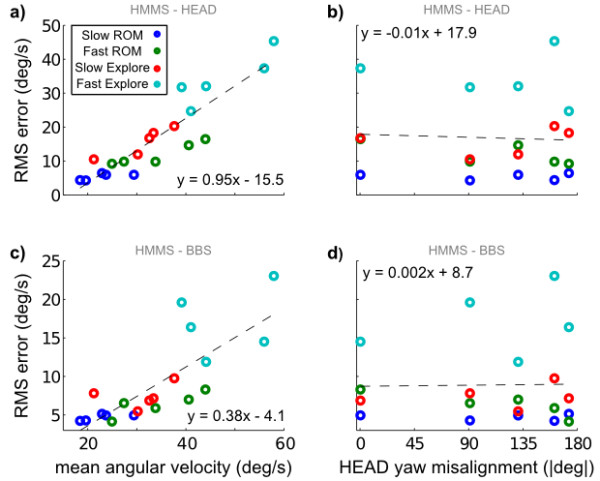
**Alignment errors for all trials and movement types**. a) Alignment error between HEAD and HMMS sensors increases nearly linearly with average angular velocity of the datasets. c) Errors between BBS and HMMS also increase linearly with average velocity, but with a 60% smaller slope. Plotting the same errors between b) HEAD and HMMS or d) BBS and HMMS vs. experimental misalignment reveals no additional trend.

Recall from the recording protocol there were multiple trials (each having different initial HMMS and BBS misalignments) and multiple movement types (each composed of different speeds and complexity). Figure 9 shows mean RMS error between aligned signals. Importantly, there was no clear trend of increasing alignment error with HMMS misalignment (Figure 9b,d). However, both the HMMS-HEAD and HMMS-BBS velocity errors increased linearly with average HEAD angular velocities (Figure 9a,c). Error for the HMMS-HEAD increased at more than double the rate of errors for HMMS-BBS with respect to average angular velocity.

Table 1 summarizes sensor alignments shown in Figure 9. Alignment error between the HMMS-BBS was comparable to the error between HMMS-HEAD during *Slow ROM *and about a factor of two lower for the other movement types. HMMS-HEAD and HMMS-BBS alignment error for all movements was similar to simulations with 40% and 25% measurement noise (see Figure 6) added respectively. (Alternatively, this error corresponds to approximately 7.7° and 5° sensor offset without any measurement noise (as in Figure 5)). The point-to-point error was relatively stable for BBS-HMMS in all segments but *Fast Explore*. However, this metric changed dramatically between segments for HEAD-HMMS. In the *Slow ROM *movement, HMMS-HEAD alignment correlation was within 5% of the upper-bound of (R^2 ^= 0.97) for our camera calibration. However, the correlation decreased substantially for the other movement types. Excluding the *Fast Explore *motion, the HMMS-BBS alignment correlation did not suffer this degradation, it remained within 5% of the *Slow ROM *values. Aligned metrics divided by unaligned metrics (i.e. do nothing) are also shown in italics - the trends are the same as the raw metrics. Additional, metrics describing the movement segments are given.

**Table 1:** Average Sensor Alignment over Segments

	Slow ROM	Fast ROM	Slow Explore	Fast Explore	All
**HEAD: HMMS**					

RMS error	5.4°/s	12.0°/s	15.6°/s	34.2°/s	17.0°/s

Point-to-point error	61.8%	36.8%	108.6%	158.5%	106.6%

Correlation (R^2^)	0.9364	0.8748	0.7143	0.5100	0.6604

*RMS error*	*33.27%*	*53.05%*	*68.86%*	*78.55%*	*66.22%*

*Point-to-point error*	*42.28%*	*60.91%*	*71.22%*	*86.74%*	*66.07%*

*Correlation (R*^*2*^*)*	*124.15%*	*124.74%*	*124.16%*	*127.57%*	*123.49%*

**BBS: HMMS**					

RMS error	4.7°/s	6.4°/s	7.5°/s	17.1°/s	8.7°/s

Point-to-point error	34.9%	40.0%	48.0%	94.1%	54.4%

Correlation (R^2^)	0.9676	0.9809	0.9391	0.8551	0.9259

*RMS error*	*26.70%*	*29.50%*	*38.42%*	*56.87%*	*40.09%*

*Point-to-point error*	*24.98%*	*30.69%*	*39.27%*	*60.40%*	*40.01%*

*Correlation (R*^*2*^*)*	*130.21%*	*127.48%*	*126.64%*	*129.71%*	*129.26%*

**Movement description**					

RMS angular velocity	22.8	34.2	31.0	47.7	33.1

RMS range	45.8	68.4	62.1	95.4	66.2

Range of velocities	233.0	434.0	191.6	292.2	467.2

Alignments are calculated within each segment (or over all segments for **All**) for each trial by randomly selecting 70% of the data in each trial for training (20 random selections) and using the remaining 30% of data for testing. Metrics were averaged over these 20 test sets, then over the three axes, then over the five trials. *Italics entries are the aligned metrics divided by the unaligned measurements for the same segments. *For example, HEAD: HMMS RMS error is reduced more than 75% by using the alignment algorithm compared to leaving the sensors unaligned.

**Figure 10:**
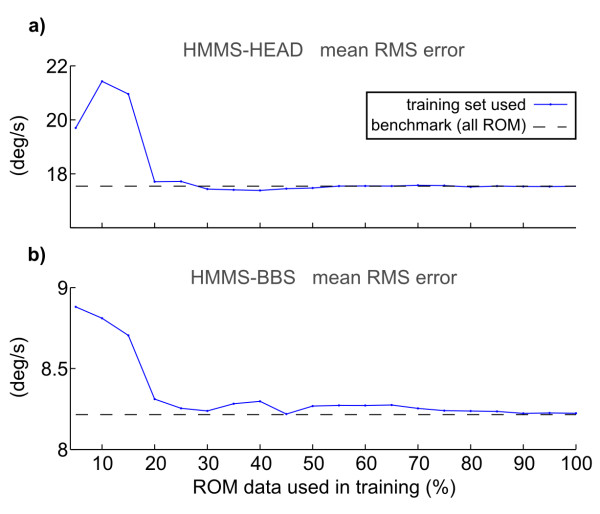
**Alignment algorithm generalization**. Both the HMMS-HEAD (top) and HMMS-BBS (bottom) reached the same levels as the benchmark (i.e. training with 100% of *ROM *data + some *Slow Explore *data) using only 25% of *ROM *data for training.

We checked alignment generalization in Figure 10. Specifically, we excluded *Fast Explore *(poor performance for both sensor pairs - likely due to slip) and concatenated the other three movement types for each trial. The *Slow Explore *segment of each trial *always *formed the *test set *(~30% of dataset). Using both *ROM *segments for *training *(~70% of dataset) yielded alignment error of 17.5°/s and 8.2°/s for HMMS-HEAD and HMMS-BBS alignments respectively (black lines in Figure 10). However, we also created smaller *training *sets (blue points in Figure 10) while maintaining the same test set with the intention of checking how much real data was necessary. For both the HMMS-HEAD and HMMS-BBS, using only the first 25% of training data resulted in similar performance as standard training, i.e. the initial 5.32 s of *Slow ROM *was sufficient to find alignment between the HEAD or BBS and HMMS. This roughly agreed with our simulations (see Figure 6b).

### Performance differences

The HMMS-BBS alignment was more accurate than HMMS-HEAD. This was surprising because the BBS was less rigidly attached to the skull and this sensor was a prototype composed of two approximately orthogonal 2D gyros, which could degrade measurement accuracy. However, HEAD measurements relied on markers that may "slip" (artificial motion of one or more markers during movement). This could strongly affect the head axis calculation used to determine angular velocity. It occurs more at higher speeds (see Figure 9) and could be reduced with additional facial markers, higher camera sampling rates, better marker tracking^iii^, improved camera calibration, and/or additional cameras.

However, there is no strong motivation to pursue such technical improvements for HEAD measurement when the BBS already performed better even as a prototype; with the important caveat that both measurement systems suffered from noise in this experiment. Additionally, the BBS requires little additional space to collect measurements (~10 cm clearance from the subject's mouth, compared to multiple cameras each at least 1 m away from the subject). To reduce the BBS measurement noise, dental impression material can be used to create a mould of the patient's teeth to increase stability of the bar, facilitate improved alignment with the skull (using the imaging technologies discussed in Methods), and significantly increase subject comfort during the calibration. (We suspect bite-bar slippage is partially responsible for HMMS-BBS alignment degradation at higher angular velocities, as the algorithm did not suffer such effects in simulations.)

## Conclusions

In this paper we proposed a straightforward optimization and protocol to align angular velocity measurements from different sensors as would be necessary in a vestibular prosthetic. The implication of this work is that vestibular prosthetic sensor could replace "natural" SCC angular velocity sensing in the semi circular canals via a three-part alignment. The three components are: a) creating a BBS aligned with HEAD; b) finding the proper **R **between HMMS-BBS; c) rotating the HMMS measurements to the BBS and then to the SCC using the HEAD-CANAL alignment.

Encouragingly, this optimization can be completed accurately with only limited data and simple sensors. We thoroughly benchmarked the optimization in a simulation environment to understand both strengths and fundamental limitations. These simulations were carefully designed to mimic realistic constraints a vestibular prosthetic may face. The optimization was insensitive to dramatically larger (50×) errors in HMMS fixation to the skull - error metrics were significantly lower for the BBS optimization compared to our approximation of *state of the art *surgical alignment.

Additionally we compared two different sensors that could be used for acute measurements of head rotations during alignment of a vestibular prosthetic sensor. We found that the simple bite-bar sensor (BBS) could be more accurately aligned with the skull-fixed sensor (HMMS) compared to motion capture estimates of HEAD rotations. This suggests that the BBS had less measurement and/or movement noise than our attempt to directly measure HEAD because the other measurement noise source (HMMS) was common. Additionally, by testing multiple movement types and velocities we confirmed the simulation results, which suggested a series of slow (for a healthy subject) range of motion movements (total time < 10 s) was sufficient for accurate sensor alignment. (Specifically, sensor alignment did not improve by including either additional Slow ROM data or data from other movement types into the training set.)

We feel that the recording protocol used for the proof-of-concept experiment (*Methods: Recording Protocol*) could be simplified such that it is more clinically relevant for two reasons. First, the theory, simulations, and actual recordings all showed that head movements required of the patient during the calibration procedure do not need to be as large as the patient will experience naturally. This will be beneficial for patients with reduced movement capabilities. Second, the data collection and processing time is very short (conservatively 10 minutes total) which should minimize patient efforts and clinician labor. Based on our results, the following protocol could be useful for clinical alignment of a vestibular sensor.

1) Align (subject-specific) BBS with HEAD coordinate and have subject bite down

2) Begin recording BBS and HMMS measurements synchronously

3) Ask subject to move head slowly to create rotations in yaw, pitch, and roll (or allow the subject to remain still and mechanically rotate them)

4) Calculate BBS-HMMS and HEAD-CANAL^iv ^alignments

5) Download appropriate rotation matrix to prosthetic

Specifically, a patient might have a vestibular prosthetic implanted on day 0 and recover from the surgery and/or adapt to baseline electrical stimulation for *N *days [18]. On day *N*+*1*, before using the gyroscope signals to modulate the stimulation rate, the patient returns to the clinic for calibration and performs the protocol above. The result is an optimized *R *matrix (based on Eqn. 4).

Downloading the *R *matrix components to the prosthetic's stimulation controller could require some further manipulations. In our prosthetic [19,20] the microcontroller does not have a Floating Point Unit, but we still achieve high correlation (R^2 ^= 1.0 for 100 rotations applied to 100 datasets, 100,000 total simulations) via bit shifting. Specifically:

1) Multiply all nine rotation matrix (*R*) elements by 2^15^

2) Round to the nearest integer

3) Download *R *integer elements into microcontroller (one time)

4) Perform alignment calculations (i.e. Eqn. 1)

5) Divide results by 2^15^

6) Repeat steps 4 and 5 for each angular velocity sample

Now the controller can convert gyroscope measurements to physiological coordinates and modulate stimulation rate using appropriate transfer functions [4,5,10,16,19].

Through our simulations, we have addressed a natural criticism to this alignment method, "why not set a proper alignment during the prosthetic implant surgery?" We achieved more accurate alignment without any of the known risks. Specifically, surgical alignment will not always be possible. Second, when possible, it is not easy to rapidly assess alignment during surgery. Even if another method (e.g., high resolution imaging) were developed that assessed alignment during surgery, our alignment method would provide a simple way to verify the new method functionally and to implement the desired realignment. Additionally, assuming accurate sensor alignment could be achieved during surgery, why spend any surgical time/effort on this when better performance can be attained with a short (circa 10 min.) post-surgery procedure? Obviously, lengthening the surgery duration to perform an in situ calibration or to align sensors with some ideal orientation increases total surgery time, which could prove detrimental to patient recovery. Finally, the natural healing process may disrupt a surgical alignment, e.g. connective tissue growth could slightly move the sensor.

The alignment protocol investigated herein could be adapted (as suggested by [14] and recently shown in [11]) such that it directly transforms the sensor signals to yield desired VOR spatial characteristics or to yield some other functional behavioural outcome (i.e., balance or psychophysical performance). While the HMMS-CANAL coordinate transformation should only be performed once (assuming no change in sensors or implant), alignment using functional metrics could be updated periodically. This updating would create an asynchronous co-adaptation of the vestibular prosthetic and the user's brain. Such co-adaptation may yield both improved functional outcomes and more rapid prosthetic integration.

## Competing interests

DMM is an inventor on patents titled *Sensor Signal Alignment *[14,19] that motivated the alignment optimization. During the review process of this manuscript, Massachusetts Ear & Eye Infirmary established licensing and research agreements with MedEl (Innsbruck, Austria). The licensing agreement includes licensing of the sensory signal alignment patents [14,19]. MedEl did not provide funding for this research. The authors declare no other competing interests.

## Authors' contributions

JD participated in the study design, developed the alignment method, participated in bite-bar design, performed the human experiments, performed all post-processing and analysis, and drafted the manuscript. JC participated in bite-bar gyro design and MTx integration into human experiments. SM participated in study design, coordinated the study, and provided manuscript feedback. DMM conceived of the alignment method, participated in study design, refined the angular velocity calculations, evaluated study results, and helped draft the manuscript. All authors read and approved the final manuscript.

## Endnotes

^i ^This research was done in the context of the CLONS project, which aims to create a closed-loop prosthetic that interfaces directly to vestibular neurons - bypassing dysfunctional semi-circular canals (CLONS details in [20]). CLONS is an acronym for CLOsed-loop Neural prosthetics for vestibular disorderS.

^ii ^The rotation order in Eqn. 2 can be directly related to Figure 1. The rotation involves initially rotating the frame about the *Z*-axis by *yaw*, then rotating about *X' *by *roll*, then rotating about *Y'' *by *pitch*. The ' and '' indicate that the first and second rotations affect the axis positions before the rotations occur.

^iii ^The authors were not experts in motion capture. Although we operated the system correctly, a more skilled Vicon operator likely could reduce marker slip frequency.

^iv ^The final alignment step is to translate the BBS approximation of HEAD angular velocity into CANAL coordinates. This could be achieved as we described in *Methods-Coordinate Systems *and *Results-Simulations*. Patient-specific HEAD-CANAL alignments could also be calculated with individual CT scans [16]
